# Effects of methods of descending stairs forwards versus backwards on knee joint force in patients with osteoarthritis of the knee: a clinical controlled study

**DOI:** 10.1186/1758-2555-2-14

**Published:** 2010-06-11

**Authors:** Masaki Hasegawa, Takaaki Chin, Sadaaki Oki, Shusaku Kanai, Koji Shimatani, Tomoaki Shimada

**Affiliations:** 1 Department of Physical Therapy, Faculty of Health and Welfare, Prefectural University of Hiroshima, 1-1 Gakuen-cho, Mihara City, Hiroshima 723-0053, Japan; 2 Department of Rehabilitation Science, Kobe University Graduate School of Medicine in Hyogo Rehabilitation Center, 1070 Akebono-Cho, Nishi-Ku, Kobe City, Hyogo 651-2181, Japan; 3 Department of Orthopedic Surgery, Kousei General Hospital, 2-5-1 Enichi-cho, Mihara City, Hiroshima 723-8686, Japan; 4 Department of Rehabilitation Science, Kobe University Graduate School of Medicine, 7-10-2 Tomogaoka, Suma-ku, Kobe City, Hyogo 654-0142, Japan

## Abstract

**Background:**

The aim of this study was to investigate the kinetic characteristics of compensatory backward descending movement performed by patients with osteoarthritis of the knee.

**Methods:**

Using a three-dimensional motion analysis system, we investigated lower extremity joint angles, joint moments, joint force of the support leg in forward and backward descending movements on stairs, and joint force of the leading leg at landing in 7 female patients with osteoarthritis of the knee.

**Results:**

Compared with the forward descending movement, knee joint angle, joint moment and joint force of the support leg all decreased in the backward descending movement. Joint force of the leading leg at landing was also reduced in the backward descending movement. In addition, we confirmed that the center of body mass was mainly controlled by the knee and ankle joints in the forward descending movement, and by the hip joint in the backward descending movement.

**Conclusions:**

Since it has been reported that knee flexion angle and extensor muscle strength are decreased in patients with osteoarthritis of the knee, we believe that backward descending movement is an effective method to use the hip joint to compensate for these functional defects. In addition, due to the decreased knee joint force both in the leading and support legs in backward descending movement, the effectiveness of compensatory motion for pain control and knee joint protection was also suggested.

## Background

Patients with osteoarthritis of the knee (OA patients) have limitations in motion in various daily activities due to functional defects, which include pain, reduced range of motion and decreased strength of the muscles surrounding the knee joint [1-3]. In particular, motions requiring deep flexion of the knee joint are commonly limited, and ascending and descending movements often become challenging [4]. Compared with the ascending movement, far greater muscle contraction is needed to control forward motion of the body when descending stairs due to an increased knee joint flexion angle [5].

Although OA patients are advised to avoid ascending and descending stairs, learning a safe and comfortable method of using stairs is still important since stairs cannot be avoided in many houses and public facilities in Japan. In clinical practice, therefore, the two-feet-one-step descending stairs method using the affected side as the leading leg is frequently recommended in order to relieve stress on knee joints. Since both lower extremity involvement is common in OA patients, it is important to take into consideration protection of the knee joint on the support leg side as well as the burden of the leading leg.

Focusing on the backward descending movement, which is a compensatory movement for pain control and knee joint protection employed by OA patients, we previously investigated the mechanics of this movement in healthy subjects and confirmed that control of the body mass was mainly done by the knee and ankle joints in the forward descending movement and by the hip joint in the backward descending movement with a decrease in knee joint flexion angle and joint moment [6,7].

Although Beaulieu et al. previously reported similar results in the changing patterns of the knee joint angle and moment [8], all of the subjects they evaluated were healthy adults, and the knee joint was not the main focus of their research. In the present study, we compared joint stress in each stair descending method by evaluating the joint force generated during forward and backward descending movements in OA patients.

## Methods

### Subjects

The subjects included 7 female volunteer OA patients (age: 68.4 ± 7.8 years; height: 151.2 ± 8.4 cm; weight: 61 ± 7.8 kg; mean ± standard deviation). All subjects were diagnosed with bilateral osteoarthritis of the knee and had a favorable course with conservative management. The grade determined by the Modified Kellgren and Lawrence Scale for all subjects was 1 or 2. The Japanese Orthopedics Association grade was 85.7 ± 6.7 for the right side and 80.0 ± 5.0 for the left side. The passive knee joint flexion angle was 130.0 ± 10.0° for the right side and 126.4 ± 14.1° for the left side. All subjects were able to walk independently.

Prior to the start of the study, a detailed description of its purpose was provided and informed consent was obtained. The present study was performed in accordance with the Declaration of Helsinki and the protocol was approved by the Ethics Committee of Kosei General Hospital.

### Experimental setting

**Figure 1:**
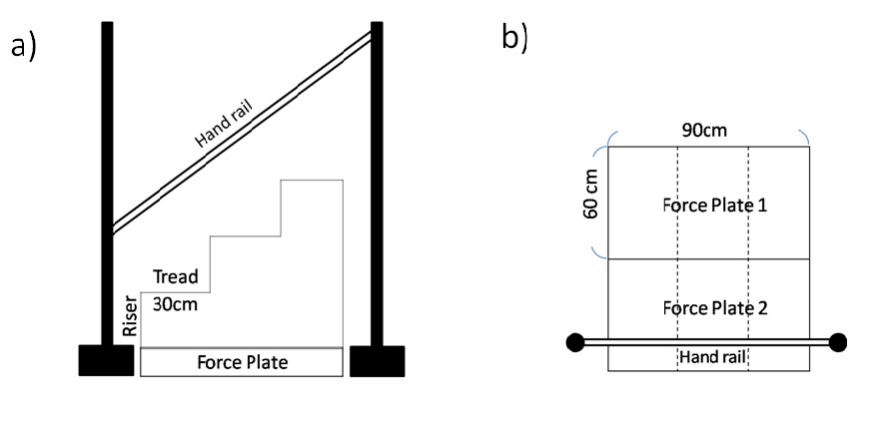
**Experimental setting**. a) Side view b) Plane view (measurement for right osteoarthritis).

Stairs which all of the 7 OA patients could ascend were constructed in order to measure various movement parameters during forward descending (FD) and backward descending (BD) movements. The stairs used in the present study had three steps with a tread width of 30 cm and a riser height of 10 cm, 15 cm or 20 cm. The stairs were made to match the size and the height of the force plate placed beneath the staircase, which consisted of right and left parts (Figure 1). During the experiment, a non-slip mat was placed on the force plate so that none of the stairs were in contact with each other [9]. The force plate level was reset to the zero base line to eliminate the weight of the stair.

In addition, a handrail was installed beside the force plate to prevent falls and to secure the safety of the subjects, who were allowed to lightly touch it in case of pain or instability (but using it for pushing and pulling was not allowed). The handrail was mounted at the same level as the subject's greater trochanter and 10 cm away from the body.

### Experimental apparatus and tasks

This study used a 3-dimensional motion analysis system comprising a VICON512 infrared position sensor (6 cameras; Oxford Metrics, UK) and a force plate (Kistler, Switzerland) with sampling frequencies set at 120 and 1080 Hz.

Infrared reflection markers were attached to 12 areas on each subject: top of the head; left and right acromion; left and right greater trochanters; lateral epicondyles of both knees; left and right lateral malleoli; left and right fifth metatarsal heads; and left inferior angle of the scapula (left/right-differentiation markers).

Each subject was asked to start in a static standing position at the top of the stairs, and began descending on a signal. The subject performed either FD or BD movement at a self-selected speed to reflect the subject's natural descending pattern. For safety reasons, the risers were installed starting from the lowest riser in the order of height and the subjects were instructed to first perform the FD movement. Three measurements were made under each condition. Each time riser height was changed, and a rest interval of about 3 min was provided. The study was conducted barefoot in order to mirror normal activities in Japanese homes.

The subjects were also given a written questionnaire to determine their preference in the descending method.

### Data extraction and interpretation

**Figure 2:**
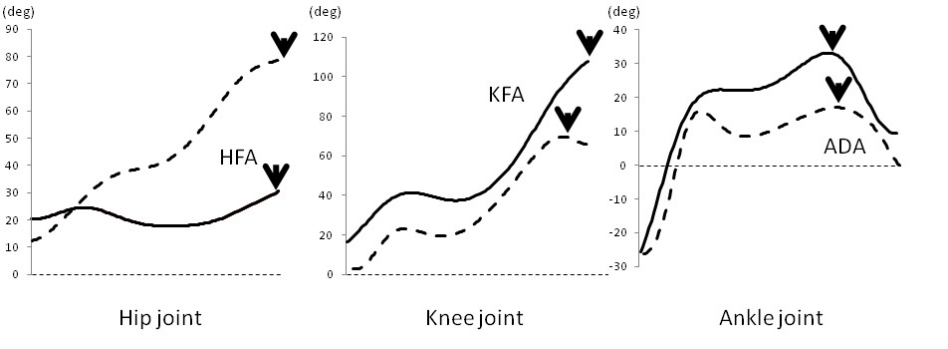
**Data extraction points for hip flexion angle (HFA), knee flexion angle (KFA), and ankle dorsal flexion angle (ADA)**. Solid line indicates forward descending movement, dotted line indicates backward descending movement, and arrows indicate data extraction points.

**Figure 3:**
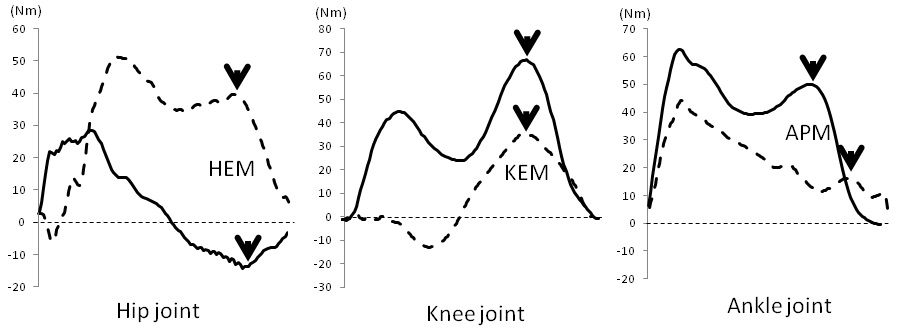
**Data extraction points for hip extension moment (HEM), knee extension moment (KEM), and ankle plantar flexion moment (APM)**. Solid line indicates forward descending movement, dotted line indicates backward descending movement, and arrows indicate data extraction points.

**Figure 4:**
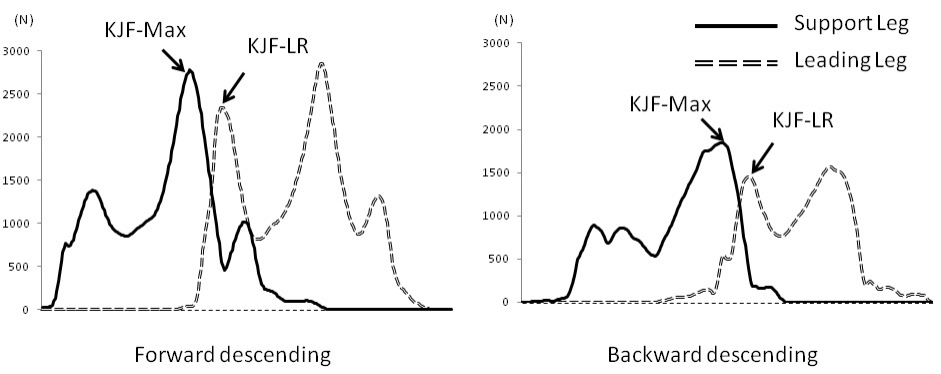
**Data extraction points for knee joint force in forward and backward descending movements**. KJF-MAX indicates joint force maximum and KJF-LR indicates joint force loading response.

ARMO software (Gsport Inc. Japan) was used for data extraction and interpretation. During the period when a subject descended the stair with the support leg holding the body weight (descending support phase), the peak values of the following data were extracted: maximum knee joint force (KJF-Max), knee flexion angle, knee extension moment, ankle dorsal flexion angle, ankle plantar flexion moment, hip flexion angle, and hip extension moment. In addition, the peak value of the knee joint force (KJF-LR) was extracted during the shock-absorbing phase when the leading leg landed (loading response phase). Joint moment and joint force were normalized to body weight. The points for data extraction during FD and BD movements for angle are shown in Figure 2, for moment in Figure 3, and for joint force in Figure 4.

While the leading leg was defined as the affected leg in this study, for patients with bilateral osteoarthritis of the knee, the leg providing more comfort in the descending support phase was defined as the support leg. Based on inverse dynamics, joint force was obtained by the summation of muscle tensions generated by all muscle groups surrounding the knee joint using the Newton-Euler equations of motion [10]. Paired t-tests were conducted to compare the values extracted in both descending methods for each riser height. P values less than 5% were considered significant.

## Results

### Kinematic and kinetic factors

Detailed results are shown in additional files 1 and 2 for angle and moment, respectively, in FD and BD movements at the different riser heights.

In terms of angle, when compared to the FD movement, hip flexion angles were significantly increased (all p < 0.01) and knee flexion angles and ankle dorsiflexion angles were significantly decreased (all p < 0.01) in the BD movement.

With regard to moment, when compared to the FD movement, hip extension moments were significantly increased (all p < 0.01) and knee extension moments were significantly decreased (all p < 0.01) in the BD movement. Moreover, when compared to the FD movement, ankle plantar flexion moments were significantly decreased (p < 0.01 for 10 cm riser height and p < 0.5 for 15 and 20 cm riser heights) in the BD movement. Therefore, when compared to FD, the hip joint moment and angle increased, but knee and ankle joint moment and angle decreased during BD.

### Knee joint force

Detailed results are shown in additional file 3 for knee joint force in FD and BD movements at the different riser heights.

KJF-LRs were significantly lower for riser heights of 15 and 20 cm (both p < 0.01), but not significantly for the riser height of 10 cm, in the BD movement, as compared to the FD movement. Converted in terms of body weight, KJF-LR increased by 3.67-4.78 times in the FD movement and by 3.16-3.70 times in the BD movement, indicating a 14%-26% decrease in joint force in the BD movement.

KJF-Max values were significantly lower for all riser heights (all p < 0.01) in BD movement, as compared to FD movement. Converted in terms of body weight, KJF-Max increased by 6.31-8.89 times in the FD movement and by 3.69-5.34 times in the BD movement, showing a 40%-52% decrease in knee joint force in the BD movement. When compared to FD, knee joint force decreases during BD.

### Questionnaire

The results of the questionnaire showed that most of the subjects (n = 5) preferred descending stairs backwards to descending forwards. The subjects who preferred descending forwards (n = 2) stated that to descend backwards is difficult, because they cannot see where they are going.

## Discussion

In this study, we found that methods of descending stairs affected the range of motion and stress on joints in the lower extremities. Compares with the FD movement, the kinematic and kinetic characteristics of the BD movement compared with those of FD movement can be summarized and interpreted as follows. The results for joint angle showed that, in the descending support phase, ankle dorsiflexion angle and knee flexion angle were increased in the FD movement, whereas hip flexion angle was increased in the BD movement. Similarly, in the descending support phase, ankle plantar flexion moment and knee extension moment were increased in the FD movement, whereas hip extension moment was increased in the BD movement. These findings suggest that the main muscle group involved in controlling the body mass is different for each descending method. We therefore conclude that balance of the whole body was mainly controlled by the knee and ankle joints in the FD movement, and by the hip joint in the BD movement. At the start of the FD movement, the knee joint is flexed and the ankle joint is dorsiflexed. These motions move the centers of the knee and ankle joints away from the floor reaction force vector, which increases each joint moment. On the other hand, in the BD movement, the body mass moves towards the heel, with the floor reaction force vector passing near the center of the ankle joint and the center of the knee joint moving toward the floor reaction force vector. These series of motions decrease ankle plantar flexion moment and knee extension moment. In addition, the hip flexion in the BD movement moves the center of the hip joint forward and away from the floor reaction force vector, which increases hip extension moment.

**Figure 5:**
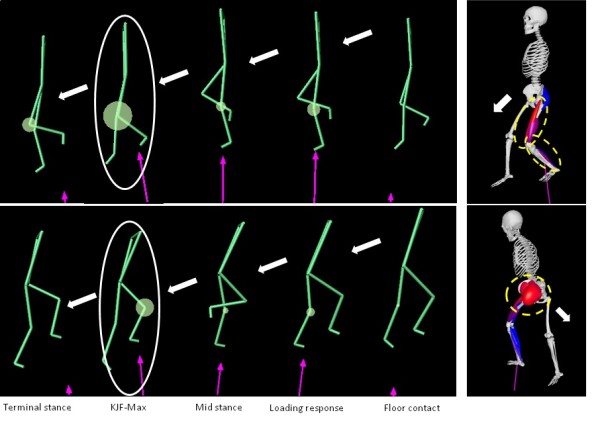
**Parallel between FD and BD**. The circles indicate KJF-MAX. The dotted-line circles indicate the strong contractions of the muscle groups at KJF-MAX. Forward descending: increased tension in the knee extensors and ankle plantar flexors occurs. Backward descending: increased tension in the hip extensors occurs.

From the results of joint moment, the joint force of the support leg can be summarized as follows. Smith et al. reported that knee joint force was influenced by the muscle tension surrounding the knee joint [11]. Consequently, increased ankle plantar flexion moment and knee extension moment in the FD movement increase the muscle tension of the knee extensors and ankle plantar flexors, which in turn generates greater knee joint force. On the other hand, in the BD movement, tension in the hip extensors increases to control the body mass, resulting in less tension in the knee extensors and ankle plantar flexors while descending, this in turn results in a decrease in knee joint force (Figure 5).

Lastly, we observed a decrease in knee joint force of the leading leg at landing in the BD movement. This is thought to be due to changes in descending control ability brought about by changes in joints and muscles in the support leg with the different descending methods. Moreover, heel-off in the FD movement narrows the base of support. This causes the center of the body mass to move in front of the base of support of the leading leg for stability. On the other hand, since the soles are completely placed on the floor during the descending support period in the BD movement, descending control on a wide base of support is possible. Therefore, the knee joint force of the leading leg at landing is thought to be reduced in the BD movement, where enough support for descending control is secured.

### What is the significance of backward descending movement in OA patients?

Decreased knee flexion angle and muscle strength of the knee extensors have been reported in OA patients [12]. In addition, osteoarthritis of the knee is more common in the elderly, who have been reported to have decreased postural control from poor use of the ankle joint due to reduced dorsiflexion angle and plantar flexor strength [13]. The FD movement, in which stress on the ankle and knee joints increases, is thought to be undesirable for OA patients. In contrast, the BD movement in which stress on the ankle and knee joints decreases is recommended for OA patients with decreased function of the knee and ankle joints.

BD movement with decreased joint force is thought to be effective from the standpoint of joint protection. Jensen et al. reported that climbing stairs and squatting were among the risk factors for developing osteoarthritis of the knee because of increased loading on the tibiofemoral joint [14]. Climbing stairs and squatting have been thought to increase loading due to the larger knee extension moment and joint force generated with an increasing knee flexion angle [15-17]. Moreover, an increased knee flexion angle has been reported to reduce the tibiofemoral contact area and loading surface of the menisci, leading to an increase of the load/contact area ratio [18-20]. In the present study, knee extension moment and joint force of the support leg (KJF-Max) were also increased in the FD movement because of the greater knee flexion angle and higher riser. Since excessive loading on the articular surface is thought to be associated with microscopic injuries of the knee joint cartilages, progression of osteoarthritis of the knee and development of pain, these findings indicate the risk of the FD movement. On the other hand, the BD movement was may be an effective method for pain reduction and knee joint protection, since it decreases the knee joint flexion angle and joint force in the leading and support legs.

## Conclusions

We confirmed that the BD movement reduced stress on the knee joint of the support leg as well as decreased knee joint flexion angle, knee extension moment and peak joint force. Knee joint force was also decreased in the leading leg. Therefore, the BD movement on stairs is an effective method for reducing joint stress in both the leading and support legs in OA patients.

## List abbreviations

We abbreviated OA: osteoarthritis; as FD: forward descending; as BD: backward descending; as KJF: knee joint force; as and LR: loading response; as.

## Competing interests

The authors declare that they have no competing interests, no proprietary, no financial, no professional or other personal interest of any nature or kind in any product, service and/or company that could be construed as influencing the position presented in, or the review of, the manuscript entitled, "Effects of methods of descending stairs on knee joint force in patients with osteoarthritis of the knee".

## Authors' contributions

M H, conceived and designed the research, performed the experiment and wrote the article.

T C, interpreted the data and was an advisor for the research design.

S O, helped acquire the data and article construction.

S K, helped acquire the data and article construction.

K S, helped acquire the data and article construction.

T S, supervised the whole project and gave the final approval of this version to be published.

All authors read and approved the final manuscript.

## Supplementary Material

Additional file 1 **Results - Angle-**. FD: Forward descending, BD: Backward descending. HFA: Hip flexion angle. KFA: Knee flexion angle. ADA: Ankle dorsal flexion angle. Click here for file

Additional file 2 **Results -Moment-**. FD: Forward descending, BD: Backward descending. HEM: Hip extension moment. KEM: Knee extension moment. APM: Ankle plantar flexion moment. Click here for file

Additional file 3 **Results -Joint Force-**. FD: Forward descending, BD: Backward descending. KJF-LR: Knee Joint force loading response. KJF-Max: Knee Joint force maximum. Click here for file
