# Supplementary figures and images for: Effects of methods of descending stairs forwards versus backwards on knee joint force in patients with osteoarthritis of the knee: a clinical controlled study
Source: Sports Med Arthrosc Rehabil Ther Technol. 2010 Jun 11;2:14. doi: 10.1186/1758-2555-2-14 (PMC2894014; doi:10.1186/1758-2555-2-14)

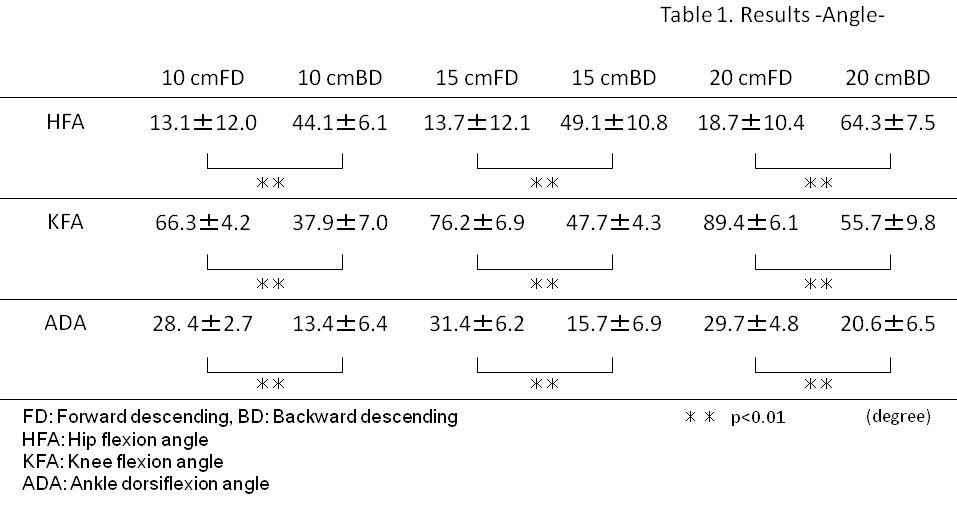

Supplement: Additional file 1 — Results - Angle-. FD: Forward descending, BD: Backward descending. HFA: Hip flexion angle. KFA: Knee flexion angle. ADA: Ankle dorsal flexion angle. [file 1758-2555-2-14-S1.JPEG]

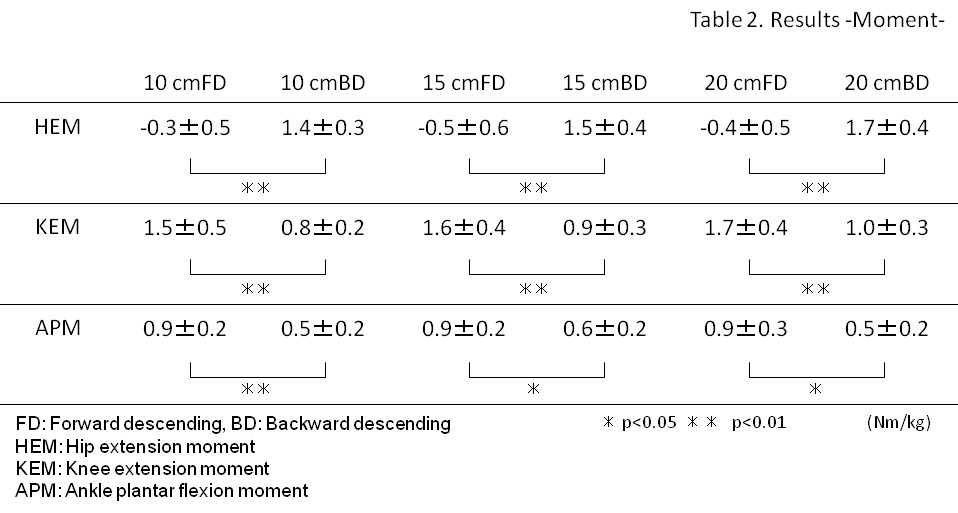

Supplement: Additional file 2 — Results -Moment-. FD: Forward descending, BD: Backward descending. HEM: Hip extension moment. KEM: Knee extension moment. APM: Ankle plantar flexion moment. [file 1758-2555-2-14-S2.JPEG]

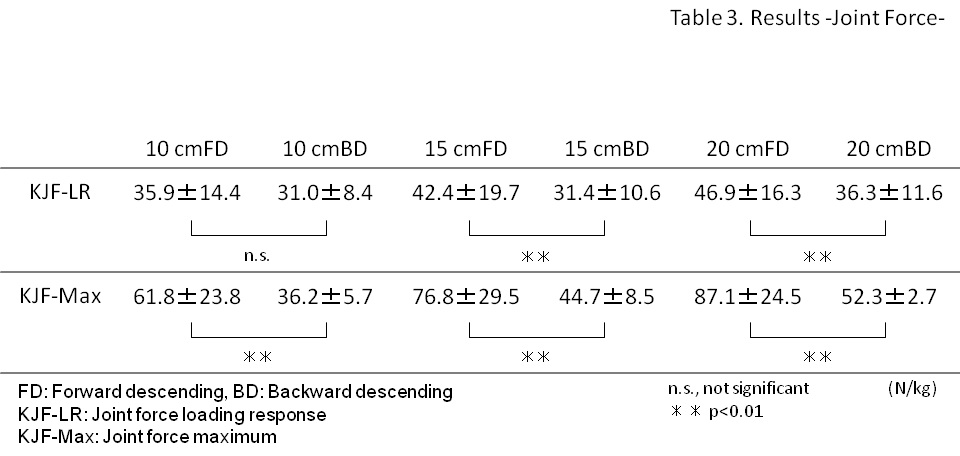

Supplement: Additional file 3 — Results -Joint Force-. FD: Forward descending, BD: Backward descending. KJF-LR: Knee Joint force loading response. KJF-Max: Knee Joint force maximum. [file 1758-2555-2-14-S3.JPEG]
